# Tree-ring correlations suggest links between moderate earthquakes and distant rockfalls in the Patagonian Cordillera

**DOI:** 10.1038/s41598-019-48530-5

**Published:** 2019-08-20

**Authors:** M. Stoffel, J. A. Ballesteros Cánovas, B. H. Luckman, A. Casteller, R. Villalba

**Affiliations:** 1 dendrolab.ch, Department of Earth Sciences, University of Geneva, rue des Maraîchers 13, CH-1205 Geneva, Switzerland; 2 Climatic Change Impacts and Risks in the Anthropocene (C-CIA), Institute for Environmental Sciences, University of Geneva, Bvd Carl Vogt 66, CH-1205 Geneva, Switzerland; 3 Department F.-A. Forel for Environmental and Aquatic Sciences, University of Geneva, Bvd Carl Vogt 66, CH-1205 Geneva, Switzerland; 4 Department of Geography, University of Western Ontario, Social Science Building, London, N6A 5C2 Canada; 5 WSL Institute for Snow and Avalanche Research SLF, Flüelastrasse 11, CH-7260 Davos Dorf, Switzerland; 6 Instituto Argentino de Nivología, Glaciología y Ciencias Ambientales IANIGLA, CCT-CONICET-Mendoza, Avenida Ruiz Leal s/n, 5500 Mendoza, Argentina

**Keywords:** Palaeoclimate, Environmental impact, Natural hazards

## Abstract

Earthquakes with magnitudes *M* > 7 can trigger large landslides and rockfalls at epicenter distances of up to 400 km, whereas moderate shaking (*M* = 5–7) is generally thought to result in abundant co-seismic mass movements in the vicinity of the epicenter. Although one might anticipate that large magnitude earthquakes off the Chilean coast would result in abundant rockfall in the Patagonian Cordillera, only limited research has explored this hypothesis. Here, we use tree-ring records from 63 cross-sections of century-old (103.9 ± 40.1 yr) *Nothofagus pumilio* trees to develop a calendar-dated record of small rockfall events (10^1^–10^2^ m^3^) on a talus slope located next to Monte Fitz Roy (El Chaltén, Argentina; 49°4′S, 72°57′W). The resulting rockfall record is used to infer that subduction zone seismicity at the Triple Junction and intraplate shaking around Lago Argentino almost systematically caused rockfall activity at this site, even if seismicity occurred at large distances (up to 300 km away) and with moderate intensity (*M* = 5–7). About one third of the rockfalls are triggered by factors other than earthquakes, predominantly in spring when freeze-thaw cycles occur frequently at the site. Despite the fact that seismicity is not the only trigger of rockfall activity at Cerro Crestón, at the foot of Monte Vespignani, we conclude that, in regions where topographic amplification plays a role, small rockfalls can be triggered by earthquakes of moderate intensity at large distances from the epicenter.

## Introduction

Earthquakes with magnitudes *M* > 7 can trigger large landslides and rockfalls (>10^8^ m^3^; ref.^1^) at epicenter distances (*ed*) <400 km (refs^2,3^), whereas moderate shaking (*M*  = 5–7) is thought to result in abundant co-seismic rockfall activity close to the epicenter location (≈15 km)^4^. In southern Chile, seismicity^5^ is largely driven by the subduction of the Antarctic and Nazca plates beneath continental South American lithosphere. Such interplate ruptures off the Chilean coast^6–8^ were the cause of the giant 1960 Valdivia (*M* = 9.5) and 2010 Maule (*M* = 8.8) mainshocks^9,10^ and the 2014 Iquique (*M* = 8.2) or 2015 Illapel (*M* = 8.3) earthquakes^11,12^. As a result of the earthquakes’ magnitude, one may expect landslides and rockslides to represent an important collateral hazard of seismic activity in the adjacent Andean Cordillera and a source of casualties and economic losses^13^. However, research in the region points to only small rockslides and debris slides resulting from the 1906 Valparaiso (*M* = 8.4), 1943 Illapel (*M* = 7.9) or 1985 Valparaiso (*M* = 7.8) earthquakes^14^, with very limited evidence for co-seismic falls. By contrast, shallower (<20 km focal depth) intraplate seismicity has been shown^15^ to correspond well with areas of large (<10^−1^ km^2^), Pliocene to recent rockfalls in the Chilean *Cordillera Principal* (32–34.5°S)^16^.

Despite the fact that mass wasting is ubiquitous throughout the Andes^17^, only a few regions have been analyzed systematically in terms of landslide age, triggering mechanisms, or links to tectonic activity or climate^18–21^. The *Terremoto Argentino* of October 27, 1894 (*M* = 7.8) represents a fine example of an earthquake that caused significant landslides along the *El Tigre* fault in the San Juan and La Rioja Provinces^22^. Evidence of causal linkages between Pleistocene and Holocene quakes and co-seismic rock avalanches also exists in the Northern Patagonian Andes (36–41°S) of Argentina^23^ and seismicity in the adjacent Chilean region has been suspected to be the trigger of mass-movement activity in Santa Cruz Province, Argentina^22^.

The use of very small rockfalls (10^1^–10^2^ m^3^) as paleoseismic indicators is a relatively recent development which is beginning to expand in scope and complexity^24,25^. Approaches may suffer from the inherent uncertainty in inferring a seismic origin because elimination of aseismic triggering of rockfall can prove difficult^26,27^. Similarly, paleoseismic landslide studies will primarily characterize the shaking history of a site irrespective of the earthquake source. The key factor driving co-seismic rockfall is the shaking intensity experienced by a rock or rock mass^28,29^. In the absence of such data at the location of rockfall occurrence, one might use earthquake magnitude (*M*) as a proxy for shaking intensity. Records of *M* are readily available in earthquake databases and could thus be tested in an exploratory approach. These records may be useful even though (i) *M* is a key metric to quantify impacts of earthquake shaking only at the epicenter and (ii) high-frequency seismic waves decay quickly from the source^30^. Despite these obvious limitations, and the fact that long-period seismic waves are more likely to be felt at increasingly large distances from seismic sources, Jibson^31^ argued that paleoseismic ground-failure studies could still help to improve understanding of shaking hazards.

In this study, we hypothesize that (i) subduction zone seismicity off the South American coast and intraplate shaking around Lago Argentino favour the occurrence of small (<10^2^ m^3^) rockfall activity from Southern Patagonian mountain cliffs that are unstable under non-seismic conditions, even if seismicity occurs at large distances (*ed* < 300 km) and with moderate intensity (*M* = 5–7); and that (ii) information from ring-width series of trees growing at the foot of talus accumulations can be used to infer paleoseismic activity indirectly through the tree-ring based dating of rockfalls^32,33^.

## Study Site

**Figure 1:**
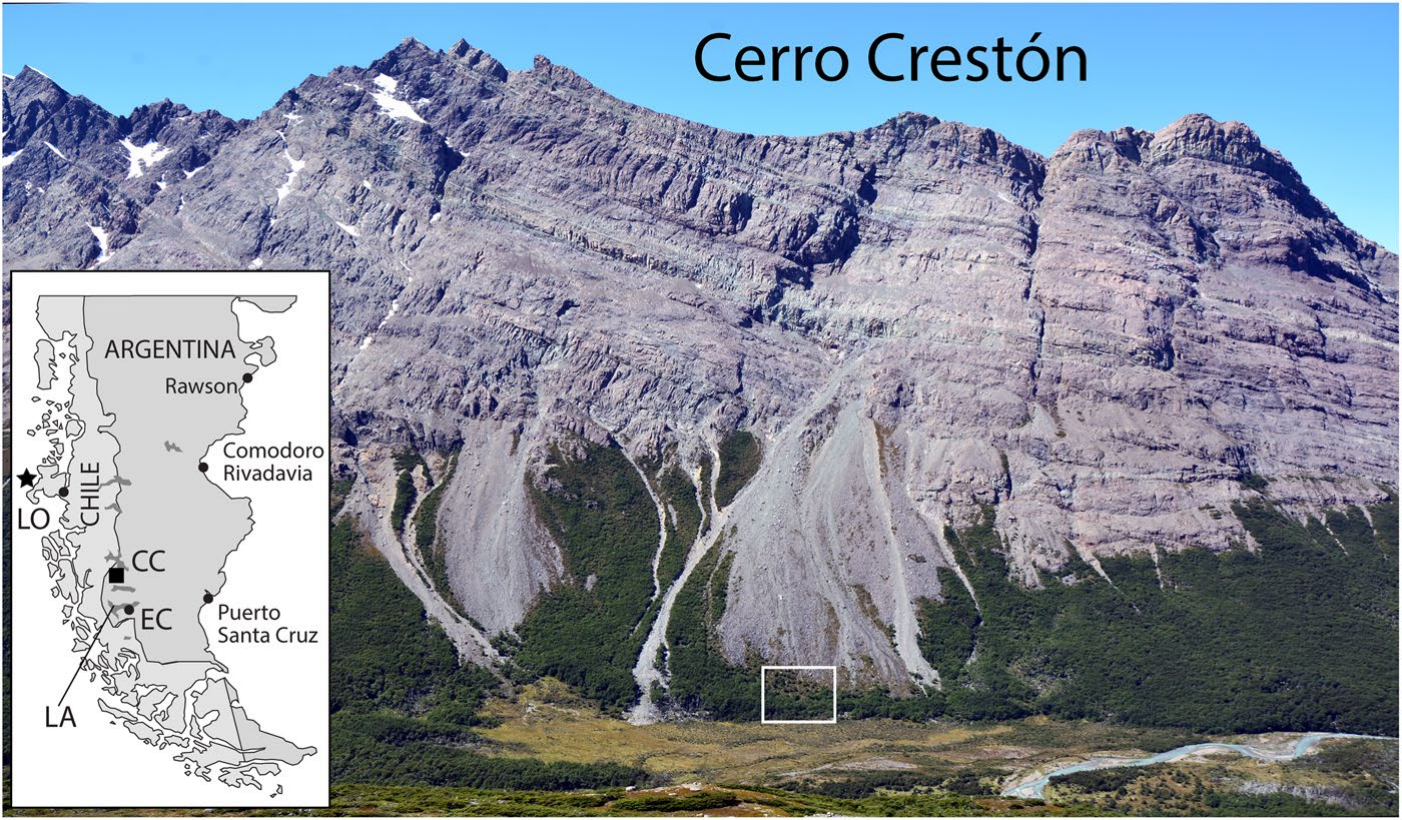
The Cerro Crestón study site is located in the Patagonian Andes, at the foot of Monte Vespignani. Fieldwork was performed at the bottom of the talus slope, where rockfall represents the principal contemporary mass movement process. Note the various, recent debris-flow deposits on either side of and above the study site. Inset map: darkest grey surfaces are lakes; CC = Cerro Crestón; EC = El Calafaté (on the shores of Lago Argentino, given as LA); LO = Liquiñe-Ofqui Fault Zone (Chile; for details see text); the star marks the Chile Triple Junction.

The talus accumulation investigated here is located near Monte Fitz Roy (El Chaltén, Argentina; 49°4′S, 72°57′W) in the Rio Toro valley (Fig. 1). Climate at the study site is characterized by a mean annual air temperature of ca. 4.8°C and precipitation totals of 1115 mm. Vegetation is dominated by open *lenga* (*Nothofagus pumilio* (Poepp. et Endl.) Krasser) forests in the lower parts of talus accumulations and on the floodplain of Rio Toro^34^.

The source of rockfalls, Cerro Crestón (1624 m asl), at the foot of Monte Vespignani (Fig. 1), is underlain by locally folded and thrusted, relatively hard, massively jointed late Jurassic volcanic sequences of dacitic and rhyolitic rocks, with scarce andesitic bodies overlying gravel sequences^35^. Rockfall locally originates from heavily weathered, disintegrated rocks on oversteepened slopes.

**Figure 2:**
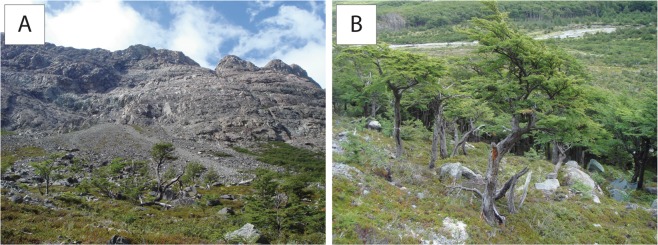
Detailed view of the study site at the foot of Cerro Crestón, Rio Toro, El Chaltén (Patagonia, Argentina). (**A**) Rockfall talus slope with upper forest fringe. **(B**) Deposits of recent rockfall activity toward the foot of the talus slope.

Rockfall is the main gravitational process at the site and has formed several talus slopes with widespread evidence of recent rockfall activity (i.e. fresh rocks on the slope surface; Fig. 2). Evidence of (recent) rockfall activity can also be found at the contact of the talus with the alluvial plain; here, the movement of rocks and boulders is abruptly stopped by the soft and often swampy surface (see Fig. 1). Evidence of runoff on the slope is diffuse. Three debris flow tracks, originating from the cliffs, run towards the base of the talus (Fig. 1). The site selected for the analysis of co-seismic rockfall activity is located at the foot of the talus slope, in a zone that is reached exclusively by the largest rockfall boulders, with no evidence of recent debris-flow activity (Fig. 2).

The most significant increase in surface seismic activity in the wider study region is observed at (i) the Chilean Triple Junction^36^, (ii) the Liquiñe–Ofqui fault system, a N-S trending intra-arc shear zone^37^, and (iii) SSE of normal faults of Lago Argentino (El Calafaté; see Fig. 1 for localization of names mentioned here). Studies on co-seismic rockfalls do not exist for the wider study area, despite the existence of supposed co-seismic landslides formed by basaltic megablocks (>10^2^ m^3^) near Monte Fitz Roy.

## Material and Methods

The use of tree rings in paleoseismology is well established and has proven successful in the reconstruction of co-seismic surface lowering^9,38,39^, mass movements^17,40,41^, and tsunamis^42,43^.

**Figure 3:**
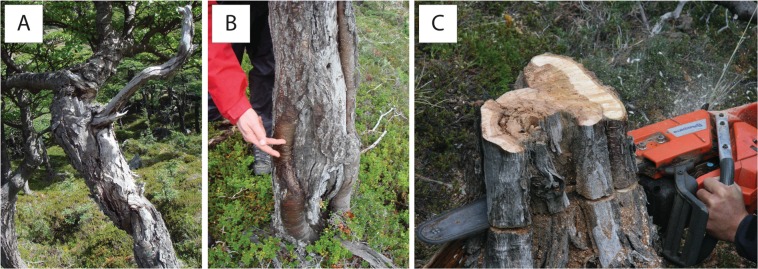
(**A**) The *Nothofagus pumilio* trees at the foot of the talus slope of Cerro Crestón show ample evidence of past rockfall damage; their stems are typically short and twisted. (**B**) Multiple scars and callus pads are seen on the stem surface. (**C**) Destructive sampling of a *Nothofagus pumilio* tree with at least three rockfall scars visible on the unprepared surface.

This study was based on tree-ring analyses and builds a comprehensive case for paleoseismology through the calendar-dating of co-seismic rockfalls. The suitability of trees in recording co-seismic falls was realized with 20 old (mean age: 103.9 ± 40.1 yrs) *N*. *pumilio* trees (Table 1) growing at the base of a talus slope at Cerro Crestón (Fig. 1) and containing ample evidence of past rockfall activity (Fig. 3). In the present case, sampling was restricted intentionally to twenty trees due to concerns in sectioning trees in this environmentally very sensitive, protected area of the Patagonian Andes. At the same time, and to maximize information contained in each of the sectioned trees, we targeted specimens with multiple scars and took several sections from each tree. With a total of 63 cross-sections and >100 rockfall scars, sample size is at a level considered adequate by other methodological papers in dendrogeomorphology^38,44–46^ and sufficient to test the suitability of trees in recording co-seismic rockfall activity.

**Table 1:** Age distribution of the 20 analyzed *Nothofagus pumilio* trees.

Stat.	First ring	Age
Max	1841	167
Min	1983	25
Mean		103.9
SD		40.1

The annual rings of trees that survived rockfall impacts were counted inwards from the bark, known from the date of collection, and cross-dated using standard dendrochronological procedures^47^. Dendrogeomorphic analysis included the whole range of growth anomalies induced by rockfalls (impact scars, growth suppression, growth release and reaction wood formation), but the focus here was clearly on the analysis and dating of rockfall scars^48^.

In a next step, the position of the scar within individual tree rings^49–51^ as well as wood anatomical features^45,52,53^ were used to date wounding with seasonal precision. By contrast to conifers, where the dating of scars is possible with up to monthly resolution^54^, sub-annual dating precision is somewhat more restricted in broadleaved trees due to differences in their genetic makeup, the absence of tangential rows of traumatic resin ducts (or TRDs) and a less marked transition from earlywood to latewood. Here we distinguished three sub-annual positions within tree rings of *N*. *pumilio*: (i) scars formed at the precise limit between two rings are attributed to dormancy (D), i.e. the time window after the completion of the previous year and the start of subsequent ring formation. At the study site, based on dendrometer data from the Instituto Argentino de Nivología, Glaciología y Ciencias Ambientales (*IANIGLA*) obtained ca. 700 m west of the study site, this period comprised the austral winter and is estimated to last from April to September (AMJJAS); (ii) Scars found in earlywood (E) of a growth ring corresponded to impacts inflicted between early October and January (ONDJ), whereas (iii) damage in the latewood (L) portion of a tree ring was caused by rockfalls occurring in February and March (FM).

In the case of simultaneous, multiple impacts in the same tree, only one event has been taken into account so as to avoid overestimation of activity^47,55^. Rockfall activity was considered high during years where the rockfall rate exceeds the mean by more than one standard deviation (SD). Activity is defined as moderate if activity was above the mean.

Comparison of tree-ring records and earthquake activity was performed using the SISRA Andean^56^ and the US Geological Survey National Earthquake Information Center (USGS-NEIC) databases. Systematic information on earthquake magnitude is available in these databases since 1973. Prior to this date, magnitudes can be found on the Internet for selected events, typically of larger magnitudes. For the sake of consistency, analysis therefore relies on distance control alone for the period 1940–1972.

The focus of the analysis for events occurring between 1973 and 2008 was on epicenter distances *ed* ≤ 300 km from Cerro Crestón, and magnitudes *M* ≥ 5. Only in a subsequent step did we also consider epicenter distances *ed* ≤ 500 km, so as to complement the assessment of co-seismic rockfalls and for the analysis distances (in km) affected by rockfalls (or landslides) as a function of earthquake magnitude *M* as defined by Keefer^2^.

## Results

The 63 cross-sections contained evidence of 127 growth disturbances induced by past rockfall activity (Table 2). In a vast majority of the cases (104, 81.9%), evidence was in the form of impact scars. In addition, we also observed strong growth (12.6%) increases after injury, mostly around the wounds and in the healing callus pad. Suppressed growth as a result of reduced vitality/photosynthesis was much scarcer (3.9%) and only observed in five cross sections. The formation of tension wood in trees tilted by the impact of rocks was present in two cases (1.6%). The oldest scar dated back to 1908, but because of the small number of trees available for analysis at the turn of the 20^th^ century, analysis was limited to the period AD 1940–2008 for which 97 scars could be analyzed.

**Table 2:** Growth disturbances identified in the 63 cross sections.

Type of disturbance	Count	%
Injury	104	81.9
Growth increase	16	12.6
Growth suppression	5	3.9
Tension wood	2	1.6
Total	127	100

**Figure 4:**
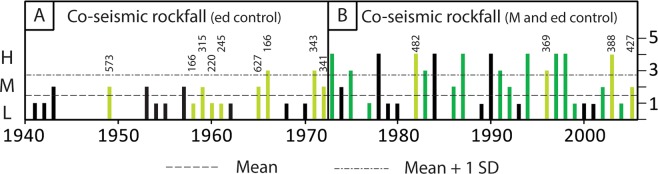
Reconstruction of rockfall activity at Cerro Crestón since AD 1940. Green bars indicate years for which the timing of earthquake activity matches with the seasonality of scars (dormancy, earlywood, latewood; for details see text and Table 3) in *Nothofagus pumilio* trees. (**A**) Comparison of rockfall rate with records of the SISRA Andean Earthquake database (Askew and Algermissen, 1985). Co-seismic rockfall events are indicated in light green, with data on the distance of the Cerro Crestón study site from the epicenter of the earthquake. Note that magnitude data are not available in the SISRA database. (**B**) Comparison of rockfall rates with the USGS/NEIC database (available since 1973). Dark green bars represent co-seismic rockfalls following quakes with M > 5 and a radial distance from the epicenter <300 km. The four years shown in light green correspond to M > 5 earthquakes but with epicenter distances >300 km. H = mean rockfall rate + 1 SD, M = mean rockfall rate; L = mean rockfall rate – 1SD.

Figure 4 illustrates the reconstructed rockfall rate at the foot of Cerro Crestón for the period AD 1940–2008. Rockfall activity is recorded in 47 years (70%) of which 27 correspond to years with seismic activity. For these 27 years, the intra-annual position of the 65 impact scars systematically agrees with the timing of seismic activity (Table 3) and thus suggests a possible causal relation and common mechanism between the two processes. For the period 1973–2008, for which information on earthquake magnitude and epicenter locations are available from the global USGS database, conditional probabilities indicate that *M* ≥ 5 earthquakes with *ed* < 300 km would have triggered moderate (M) to large (L) rockfalls in 75 and 89% of the cases, respectively.

**Table 3:** Dates of Patagonian earthquakes, their epicenter locations and magnitudes as well as distances to Cerro Crestón.

Earthquake			Coordinates		M	Distance (in km)	Rockfall (scars)	Intra-seasonal dating		Influence of snow and ice
Y	M	D	Lat	Long				Position	Season	
1949	12	17	−51.9	−72	7.8	573	2	E	ONDJ	
1959	4	8	−50.5	−73	6.1	181	1	L	FM	
1959	9	4	−47	−75	?	315	2	D	AMJJAS	likely
1960	5	24	−50.5	−74	6	220	1	D	AMJJAS	likely
1960	5	25	−47	−75	6.8	315	?	?	?	likely
1960	6	2	−46.5	−74	?	315	?	?	?	likely
1961	8	1	−47.2	−73.9	?	245	?	D	AMJJAS	likely
1961	12	9	−50.9	−73.6	6.7	240	1	E	ONDJ	
1965	11	28	−43.4	−72.1	6.2	627	2	E	ONDJ	
1966	9	29	−50.5	−72.4	5.2	166	3	E	ONDJ	
1972	2	9	−51.8	−74.1	6	343	3	L	FM	
1972	8	13	−51.8	−73.9	?	341	2	D	AMJJAS	likely
1973	5	2	−48.9	−75.8	5.2	281	4	D	AMJJAS	likely
1975	4	25	−47.7	−75.3	5	286	3	D	AMJJAS	likely
1977	5	12	−46.4	−73.9	5	327	1	D	AMJJAS	likely
1982	5	14	−45.4	−75.5	5.8	482	4	D	AMJJAS	likely
1983	8	1	−48.3	−75.9	5.6	294	3	D	AMJJAS	likely
1986	9	10	−50.2	−71.3	5	147	2	D	AMJJAS	likely
1987	6	29	−51.7	−72	5	304	4	D	AMJJAS	likely
1992	1	25	−50.4	−72.2	5.1	158	3	E	ONDJ	
1992	7	30	−50.4	−72.1	5.5	151	2	D	AMJJAS	likely
1995	2	7	−48	−75.5	5.3	283	4	E	ONDJ	
1997	4	21	−48.9	−75.8	5.2	277	4	D	AMJJAS	likely
1998	10	9	−47.7	−75.6	5.2	305	4	E	ONDJ	
1999	5	29	−50.4	−76	5	325	1	D	AMJJAS	likely
2002	11	9	−48.2	−75.5	5.6	275	2	E	ONDJ	
2003	9	12	−51.6	−75.6	5.6	388	4	D	AMJJAS	likely
2004	8	30	−50.7	−72.1	5.1	188	1	D	AMJJAS	likely
2007	4	25	−45.3	−72.7	6.2	427	2	D	AMJJAS	likely

D = dormancy of *N*. *pumilio* trees lasting locally from April thru September (AMJJAS); E = Earlywood formation between October and January (ONDJ); L = Latewood formation in February and March (FM).

Earthquake data are compared to the rockfall activity and intra-annual position of 97 scars found in 63 cross-sections of 20 *N*. *pumilio* trees. In cases where magnitude (M) information is missing a “?” was placed in the table. In the case of the earthquakes in 1960 and 1961, a clear attribution of rockfall scars to individual shaking events was not possible. Seasonal effects on rockfall processes during the winter season influence the detection of earthquake-rockfall relationships and potentially weaken results (for details see text).

**Figure 5:**
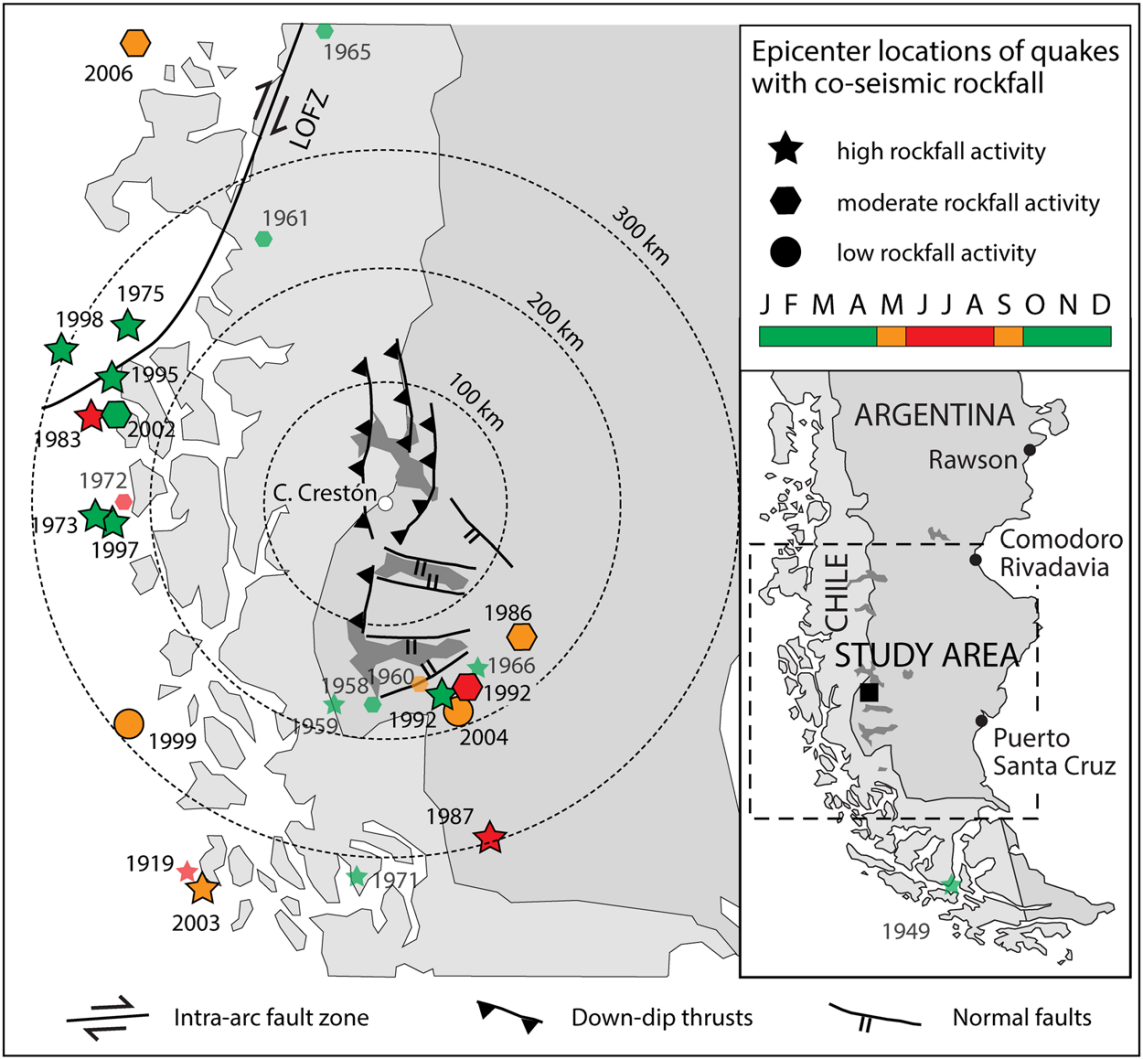
Epicenter locations of earthquakes triggering co-seismic rockfalls at Cerro Crestón. Green symbols indicate shaking events in the release area of rockfalls outside the Austral winter. Red and orange symbols indicate co-seismic winter rockfalls when the presence of ice “cementing” interstitial rock joints and snow on the talus likely reduces the (i) release and/or (ii) spread and reach of rockfalls. Smaller, semi-transparent symbols and corresponding years are for the period 1940–1973 for which epicenter distance control is available but information on magnitudes (*M*) does not systematically exist and may not be of the same quality as the one for the more recent events. LOFZ = Liquiñe-Ofqui Fault Zone.

Figure 5 illustrates epicenter locations, the intra-annual timing of seismicity and corresponding rockfall activity over the past 70 years. It seems that the response and degree of rockfall activity depends on the intra-annual timing of earthquakes, with a somewhat weaker agreement between seismicity and rockfalls during austral winters (Table 3). This weaker correspondence of rockfall rates following winter earthquakes can likely be explained by (i) the ice cement in interstitial fissures preventing the release of unstable rock masses; and by (ii) the dampening and breaking effects of the winter snow cover on the spread and downslope reach of rockfalls and the resulting deposition of co-seismic rockfalls above the forest fringe. If seismicity during austral winters is neglected, seismic records explain 89% and 100% of the moderate (M) and large (L) co-seismic rockfalls, respectively, between 1973 and 2008.

Figure 4 also shows that one-third of the impacts recorded in the *N*. *pumilio* trees (32 scars) occurred in years without earthquake activity, of which 63% were attributed to the dormant season of trees, i.e. to austral winter. Interestingly, the significant rockfall activity in 1978, 1984 and 1990 (with 4 scars each) were spring and summer rockfall events that occurred early in the growing season (1978 and 1990) or at different times of the austral summer (1984). In the case of the rockfall activity recorded in years without earthquake activity, an identification of triggers proved difficult and we could not find any significant correlations between climatic variables (temperatures, rainfall, snow) and the reconstructed rockfall activity (data not shown).

**Figure 6:**
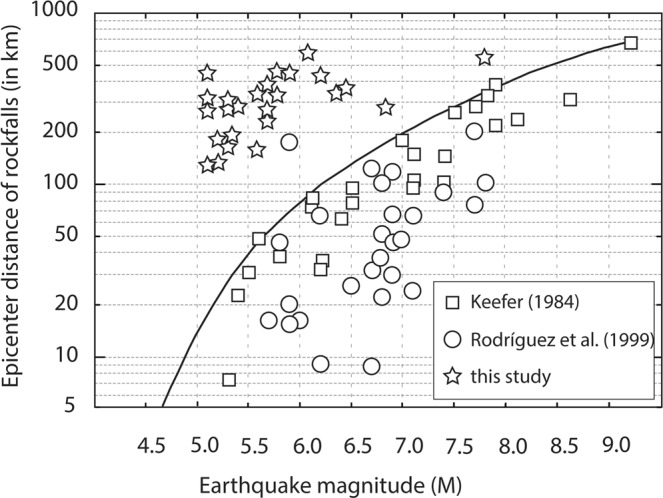
Distance affected by landslides (km) as a function of earthquake magnitude *M*. The solid line is the upper bound determined by Keefer (1984). In addition to the events for which the USGS database provides magnitude information (1973–2008), we also plot here those events identified in Table 3 for which magnitude information could be found on the Internet.

Figure 6 illustrates the distance (km) over which sites are normally affected by landslides as a function of earthquake magnitude *M*. The solid line represents the upper bound as determined by Keefer^2^. Rockfalls are generally known to occur at larger distances from epicenters than landslides and to be initiated by weaker earthquakes. As such, it may not be surprising that the maximum distances at which rockfalls can occur during *M* ≥ 5 quakes exceed those reported for landslides, and that thresholds may need to be revisited, at least insofar as small, composite rockfalls are concerned.

## Discussion and Conclusions

Rockfalls are the most abundant type of slope movement induced by seismic activity and are most common on slopes steeper than 40° with rocks that are weakly cemented or/and have closely spaced joints^2^. The rock faces of Cerro Crestón meet this description and are unstable under non-seismic conditions. Trees growing at the foot of rockfall talus accumulations at the study site are frequently impacted by rockfall and therefore can preserve evidence of co-seismic rockfall activity. Assuming that rockfalls during austral springs and summers are otherwise predominantly triggered by freeze-thaw cycles and thunderstorms, comparison of the intra-seasonal timing of rockfalls estimated by dendrochronology with the calendar dates for Southern Patagonian earthquakes is very striking (Table 3), and points to the potential for dendrogeomorphic time-series of rockfall activity to complement records of past seismic events. The fact that trees may record multiple events during their lifetime (and within the same year^57–59^) can outperform the contribution of lichenometry where the frequency of reworking of rockfall deposits and the covering of deposits by new incoming blocks may blur evidence of past events. In this study, it was possible to distinguish co-seismic rockfalls at different times in the same year in 1959, 1961, 1972 and 1992.

The plotting of distances from the rockfall source to earthquake epicenter locations also confirms the practical lower-bound seismicity for small rockfalls and earthfalls as defined by Keefer^2^ (*M* = 4) or Jibson^31^ (*M* = 5–6) as compared to larger landslides. At the same time, however, it seems that the triggering of small rockfalls from marginally stable slopes has been underestimated in previous global assessments and that the upper distance bound of co-seismic rockfalls should be revisited for small, localized events.

Figure 6 illustrates that radial distances from earthquake epicenters at which gravitational processes are triggered are roughly one order of magnitude higher at Cerro Crestón than in those cases presented in the literature^2,60^. In their assessments of impacts of moderate- and low-magnitude seismic activity in Spain and Mexico, different studies^61,62^ reported that epicenter distance and area affected by co-seismic mass movements, even if small in nature, were well above the global bounds previously reported in the literature. In our case, the potentially causal relation between seismicity and rockfalls in the Southern Patagonian Cordillera suggests a triggering of rockfalls through ground acceleration, which is possibly enhanced through the amplification of ground motion on mountain tops^63^ and the geometry of the local thrust-and-fault belt structures^64^, especially in case of seismic shaking around the Chilean Triple Junction (see Fig. 5).

Although our data – as well as the paucity of available rockfall inventories for high relief terrain – precludes further generalizations, we expect that forests at the fringe of talus slopes in tectonic settings similar to those of the Patagonian Cordillera hold considerable potential for augmenting the historical record of earthquakes. Therefore, tree-ring dating can provide important information for the evaluation of seismic hazards and thereby for studies that are concerned with the analysis of regional patterns of abundance, frequency and magnitude of earthquake-generated rockfalls^65^. Foothill and mountain areas tend to be among the locations most severely affected by collateral effects of seismic activity^13^ and also may have relatively large population concentrations^66^.
